# Effect of welding fumes on the cardiovascular system: a six-year longitudinal study

**DOI:** 10.5271/sjweh.3908

**Published:** 2020-12-16

**Authors:** Tahir Taj, Anda R Gliga, Maria Hedmer, Karin Wahlberg, Eva Assarsson, Thomas Lundh, Håkan Tinnerberg, Maria Albin, Karin Broberg

**Affiliations:** 1 Occupational and Environmental Medicine, Department of Laboratory Medicine, Lund University, Lund, Sweden; 2 Institute of Environmental Medicine, Karolinska Institutet, Stockholm, Sweden; 3 Section of Occupational and Environmental Medicine, Sahlgrenska Academy at University of Gothenburg, Gothenburg, Sweden

**Keywords:** biomarker, blood pressure, cardiovascular disease, C-reactive protein, CVD, endothelial function, homocysteine, LDL, metal, occupational medicine, particle

## Abstract

**Objective:**

This study investigated whether low-to-moderate exposure to welding fumes is associated with adverse effects on the cardiovascular system.

**Methods:**

To test this, we performed a longitudinal analysis of 78 mild steel welders and 96 controls; these subjects were examined twice, six years apart (ie, timepoints 1 and 2). All subjects (male and non-smoking at recruitment) completed questionnaires describing their health, work history, and lifestyle. We measured their blood pressure, endothelial function (by EndoPAT), and risk markers for cardiovascular disease [low-density lioprotein (LDL), homocysteine, C-reactive protein]. Exposure to welding fumes was assessed from the responses to questionnaires and measurements of respirable dust in their breathing zones adjusted for use of respiratory protection equipment. Linear mixed-effect regression models were used for the longitudinal analysis.

**Results:**

Median respirable dust concentrations, adjusted for respirable protection, of the welders were 0.7 (5–95 percentile range 0.2–4.2) and 0.5 (0.1–1.9) mg/m^3^ at timepoints 1 and 2, respectively. Over the six-year period, welders showed a statistically significant increase in systolic [5.11 mm Hg, 95% confidence interval (CI) 1.92–8.31] and diastolic (3.12 mm Hg, 95% CI 0.74–5.5) blood pressure compared with controls (multi-variable adjusted mixed effect models). Diastolic blood pressure increased non-significantly by 0.22 mm Hg (95% CI -0.02–0.45) with every additional year of welding work. No consistent significant associations were found between exposure and endothelial function, LDL, homocysteine, or C-reactive protein.

**Conclusion:**

Exposure to welding fumes at low-to-moderate levels is associated with increased blood pressure, suggesting that reducing the occupational exposure limit (2.5 mg/m^3^ for inorganic respirable dust in Sweden) is needed to protect cardiovascular health of workers.

Worldwide, over 11 million welders and additional 110 million workers are exposed to welding fumes (1). In Sweden, there are 13 000 registered full-time welders (2, 3), while an additional 250 000 workers perform some welding in their jobs (3). Welding generates respirable particles and gases. Welders are mainly exposed to small particles of iron and manganese but also to particles of other metals (eg, nickel and chromium) and particles of non-metal origin (4) due to the material being welded but also the technique. The majority of primary particles in different welding aerosols have diameters ranging from 5–40 nm but they have a tendency to form chainlike agglomerates (5). By mass, welding aerosols have a broad size distribution from coarse [particulate matter (PM) 2.5–10] to fine (PM 0.1–2.5) and ultrafine (PM 0.1), with the fine and ultrafine particles generating higher levels of reactive oxygen species (6).

In the general population, acute and chronic exposure to fine particles has been associated with increased risk of morbidity and mortality from cardiovascular diseases (CVD) (7). Furthermore, recent evidence suggests that long-term exposure to PM1 and PM2.5 increases blood pressure (8, 9). Although welders are exposed to particle levels many times higher than experienced by the general public, we still lack compelling epidemiological evidence from longitudinal studies of whether welding causes CVD. However, cross-sectional and case studies have associated exposure to welding fumes with higher blood pressure (BP) (10), impaired cardiac autonomic function (11), decreased heart rate variability and ectopic heartbeats (12), as well as damage to human coronary artery epithelial cells (13).

In this study, we aimed to investigate risk markers of CVD among currently working welders and controls, in a longitudinal cohort, by combining detailed exposure assessment data with measurements of BP, endothelial function, and plasma biomarkers.

## Methods

### Study participants

**Figure 1:**
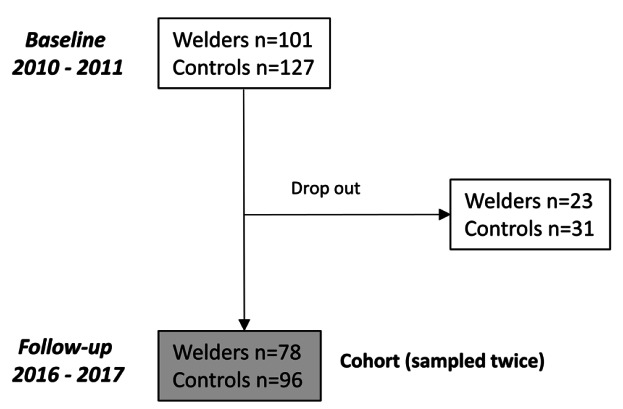
Longitudinal study design.

During 2010–2011 (timepoint 1), a total of 101 mild steel welders and 127 controls (with low occupational exposure to particles) in southern Sweden were enrolled in the study; all of them were male and non-smoking (figure 1). These welders worked in small- and medium-sized welding companies (N=10) involved in the manufacturing of forklift trucks, hydraulic lifting tables, dump trucks, asphalt rollers, heating boilers, and stoves. The controls worked mainly in warehouses with food storage and in municipalities as gardeners or janitors. Full details of the recruitment process at timepoint 1 were reported in two prior studies (10, 14).

Approximately six years later (2016–2017), a follow-up study of the welders and controls was performed [timepoint 2; see Gliga et al (15) for full description of the recruitment (15)]. At timepoint 2, welders from 9 of the 10 welding companies were included (one of the companies had closed). Of the 101 welders that participated at timepoint 1, 78 (77%) also participated at timepoint 2. Similarly, of the 127 controls that participated at timepoint 1, 96 (76%) also participated at timepoint 2. The welders and controls that did not participate at timepoint 2 either declined participation without giving any particular reason, were sick on the medical examination day, or had died since timepoint 1. Apart from welding years, which was significantly higher among those who participated at both timepoints, the baseline health and exposure characteristics did not differ between participants at timepoint 1 only (N=23) and timepoints 1 and 2 (N=78). Among the 78 welders participating in both cycles, 7 had retired or quit working due to illness since timepoint 1, and another 12 were employed at the same company as before but were no longer actively involved in welding. Those 7 who had quit working were invited to visit either their old company or the clinic for the follow-up examination. For those individuals, no respirable dust measurements or cumulative exposure data (see below) were available at timepoint 2.

### Questionnaire-based data

We used essentially the same questionnaires at timepoints 1 and 2 (with minor differences in phrasing). These were distributed to all participants one week before their medical examination and sampling. On each visit, a trained nurse checked each questionnaire for completeness and consistency of the responses. The questionnaires were used to obtain detailed job history, information about use of safety equipment (ie, personal protective equipment) and other precautions taken to avoid exposure to dust and fumes in the workplace as well as any hobbies which could entail exposure to particles (eg, from welding fumes outside of work, or diesel exhaust). The questionnaires also asked the participants about their dietary habits, physical activity, alcohol and tobacco use, education, current and past known illnesses, medication usage (prescribed by doctors or non-prescribed), and family history of CVD and other chronic diseases.

### Respirable dust measurement

Personal exposure measurement of respirable dust was performed for the active welders, and stationary area-monitoring of respirable dust monitoring was conducted for the controls. For personal sampling, a cyclone (BGI4L, BGI, Mesa Labs, USA; cut-off = 4 μm) was used for collecting respirable dust. The cyclone was fitted with a filter cassette, containing 37-mm mixed cellulose ester filters with an 0.8-μm pore size (pre-weighed) and was placed within the breathing zone of each welder. The airflow through the sampler was set to 2.2 L/min and regularly checked before, during, and after sampling with a flow meter (TSI Model 4100 Series, TSI Incorporated, USA). This personal sampling was coordinated with each company’s shift working hours and the average sampling duration was approximately 7 hours for both timepoints. Measured dust concentrations were corrected if respiratory protection was used: the measured concentration (outside respiratory protection) was divided by three as a correction factor to reflect the actual exposure level (10, 16). At timepoint 2, one welder used a half-mask, for which a correction factor of 2 was used instead (17), and another four welders used newer versions of powered air-purifying respirators with double visors, for which a factor of 50 was used [personal communication with Karlsson J-E, occupational hygienist, Clinic of Occupational and Environmental Medicine, Lund University Hospital, Sweden]. The filter samples were analyzed gravimetrically according to a validated method for determination of respirable dust. The limit of detection was set to 0.05 mg/sample.

For those welders with incomplete exposure data, their respective exposure level was estimated individually using geometric mean exposure data obtained from welders working at the same work station, engaged in similar tasks or in the same company. The use of protection devices was then corrected for as described above. For two welders, to complete missing data for the exposure assessment, exposure data previously collected at the welding companies (10, 16) were also used. Only active welders (ie, not retired or welders with non-welding work tasks) had either measured or assessed respirable dust data. In the end, there were 56 welders who had respirable dust data at both timepoints (timepoint 1: measured N = 28, estimated N = 28; timepoint 2: measured N = 46, and estimated N = 10).

For the controls at timepoint 1, full-shift personal breathing zone samples of respirable dust were collected from two companies for 19 control subjects. From four companies, area-level air pollution monitoring of respirable dust was performed using a direct reading monitor, SidePak Model AM510 (TSI Incorporated, Shoreview, MN, USA) with a Dorr-Oliver cyclone (10). At timepoint 2, stationary area monitoring of respirable dust fractions was performed using a DustTrak DRX monitor (TSI Incorporated). At both timepoints, these monitors were placed at breathing zone height in the area where workers spent the most time during their work shifts. On average, the monitoring of each control lasted approximately 4 hours at each company’s work site. In companies where workers spent time at two different workstations, measurements were taken in both areas, and a time-weighted average of the two sites was calculated.

### Calculation of cumulative exposure

For each welder, the cumulative exposure was calculated by taking the respirable dust measurement adjusted for respiratory protection and multiplying it by years of welding reported, as follows:

Cumulative exposure_1_ = respirable dust timepoint_1_ × years welding timepoint1

Cumulative exposure timepoint_2_ = cumulative exposure timepoint_1_ + (respirable dust timepoint_2_ × [years welding timepoint_2_ - years welding timepoint_1_])

We only calculated the cumulative exposure for active welders, ie, welders with measured or assessed respirable dust.

### Analysis of metals on filters from respirable dust sampling

Filters from timepoint 2 (total N=104, out of which N=46 individuals are included in this longitudinal study) were analyzed for element concentrations of Mg, Al, K, V, Cr, Mn, Fe, Co, Ni, Cu, Zn, Cd, Tl, Th, and Pb. After weighing, the filters were digested in 1 mL of concentrated nitric acid at 70 °C for 16 hours. After dilution with Milli-Q water, the metal concentrations were determined by inductively coupled plasma-mass spectrometry (ICP-MS; iCAP Q, Thermo Scientific GmbH, Dreieich, Germany) in collision cell mode, with kinetic energy discrimination, using helium as the collision gas. The detection limits were calculated as three times the standard deviation (SD) of blank filters and are as follows: 0.005 μg (Co, Cd, Tl, Pb, Th), 0.007 μg (V, Ni), 0.01 μg (Cu), 0.02 μg (Mn), 0.05 μg (Cr, Zn), 0.08 μg (Mg), 0.15 μg (Al), 0.89 μg (Fe), and 7.4 μg (K). Analytical accuracy was verified using certified reference filters in the analysis of samples, N=20 (Trace Metal on Filter Media D, Part nr. QC-TMFM-D, Lot 1530803; High- Purity Standards, North Charleston, SC, USA). The obtained versus certified mean values were 2.46 (SD 0.03), 2.45 (SD 0.04), 2.51 (SD 0.04), 2.49 (SD 0.03), and 2.51 (SD 0.03) μg *versus* 2.50 (SD 0.03) μg for V, Cr, Co, Ni, and Cu, respectively; 2.47 (SD 0.04) and 2.45 (SD 0.03) μg *versus* 2.50 (SD 0.05) μg for Tl and Pb, respectively; 1.04 (SD 0.02) and 0.98 (SD 0.01) μg *versus* 1.00 (SD 0.01) μg for Mn and Cd, respectively; 2.70 (SD 0.21) versus 2.50 (SD 0.1) μg for Fe; 2.39 (SD 0.07) μg *versus* 2.50 (SD 0.2) μg for Zn; and 49.0 (SD 0.86) μg *versus* 50.0 (SD 0.5) μg for Al. No reference values were available for Mg, K, or Th.

### Workload

There are indications that workload and occupational heavy lifting are important risk factors for CVD (18, 19). An occupational hygienist therefore roughly estimated the ergonomic workload and lifting by watching short videos (minutes) collected on the sampling days for most participants (welders and controls) at all work sites at timepoint 2. At the warehouses (controls), the workload varied greatly among work tasks, which ranged from manually moving food packaging between pallets and driving trucks from storage points to reloading points. The workload differed among the welders, but at all sites any heavy lifting was done using lifting aids. The individual workload over time was difficult to estimate reliably because the videos were too short and the tasks varied too much. An overall difference in workload between welders and controls could not be discerned; hence, workload was presumed to span a similar range at all the welders’ and controls’ companies, and it was not used as a covariate in the statistical analysis.

### Medical examination and blood sampling

A trained occupational nurse conducted the physical examinations of welders and controls, which included measurements of height, weight, BP, pulse rate, and peripheral arterial tonometry as a measure of endothelial function. At the end of each examination, blood samples were taken to assess biomarkers of risk of CVD [C-reactive protein (CRP), LDL, homocysteine]. Blood samples were left to clot at room temperature for exactly 10 minutes and then centrifuged at 2200 × *g* for 10 minutes. It should be noted that homocysteine values increase with time before separation (20) and slightly elevated values can be expected due to the 10 minutes of clotting before separation. Nevertheless, all samples were treated in the same way. After separation, plasma samples were aliquoted and kept on dry ice while they were moved to the laboratory in the Division of Occupational and Environmental Medicine at Lund University, where they were stored at -80 °C until analysis.

### Blood pressure measurement

A skilled occupational nurse measured the BP of welders and controls using a digital monitor (Boso Medicus, Bosch & Sohn GMBH, Ingelheim am Rhein, Germany) with an adjustable cuff to accommodate different arm circumferences. All measurements were performed in dim light in a silent room with comfortable seating and subjects in the supine position. For timepoint 1, one BP reading was taken from each participant; for timepoint 2, two reliable measurements were taken at 2-minute intervals and their average per participant used in later analyses.

### Peripheral arterial tonometry measurement of endothelial function

Peripheral arterial tonometry was performed during the daytime working shift. The participants’ height, weight, age, and blood pressure were first entered into an EndoPAT instrument (Itamar Medical, Caesarea, Israel). The participants were lying in a comfortable bed with both arms resting on an armrest to limit their muscle activity. The finger probes were placed on both index fingers to record the digital pulse wave amplitude (PWA) and a digital blood pressure monitor cuff was positioned on the left arm. The PWA was measured for 6 minutes until it was stable. A baseline measurement was taken for 5 minutes, after which the cuff was inflated over 200 mm Hg or to at least 60 mm Hg above the subject’s recorded systolic BP. Following the baseline recording, the cuff was deflated and a post-occlusion reactive hyperemia (RHI) signal recorded for 5 minutes. The EndoPAT software computed values for endothelial function (reactive hyperemia index, RHI), arterial stiffness (augmentation index, AI), and normalized AI at 75 beats per min (AI@;75). An RHI score <1.67 indicates impaired coronary endothelial function (21).

### Statistical analysis

For the analysis of examined participants present at both timepoints 1 and 2, we used linear mixed-effects models to evaluate the influence of being a welder (versus being a control) upon the cardiovascular outcomes of systolic and diastolic BP, EndoPAT measurements (RHI, AI@;75), and cardiovascular risk biomarkers (LDL, homocysteine, CRP). Exposure–response relationships of respirable dust, welding years, and cumulative exposure were analyzed among welders’ only in separate linear mixed-effects models analyses against the same outcomes. The mixed models included the participants as a random factor (random intercept) because they were measured twice and thus lacked independence over time, and all other predictor variables as fixed factors. Covariates included in the models were participants’ age (continuous), body mass index (BMI) (continuous), education status (from secondary school to university studies in five ordinal categories), residence (from large cities to countryside in four ordinal categories), physical activity (sedentary to intense physical in four ordinal categories), smoking (current smoker, party smoker, non-smoker), use of snus (a moist powdered form of tobacco packaged in pouches that is placed under the upper lip; yes, no), frequency of alcohol consumption (times/day/week), and frequency of vegetable consumption (times/day/week) and fish intake (≥1–0/day in seven ordinal categories). Familial history of CVD (yes, no) and use of drugs potentially affecting EndoPAT (yes, no) were added for analyzing the EndoPAT outcomes only. Pearson standardized residuals were visually inspected for evaluating model assumptions of normal distributions and equal variance (homogenous). We conducted a sensitivity analysis where we used BMI from timepoint 1 to adjust for at both timepoint 1 and timepoint 2, to test whether BMI at timepoint 2 could be an intermediate between exposure at timepoint 1 and outcomes at timepoint 2. There was no major difference in the estimates or significance levels, which indicates that the main analysis was only adjusted for potential confounders and not intermediates.

All statistical analyses were carried out in the R platform, version 3.4.3, with the *lme* package used to fit the linear mixed models. The variance explained by fixed factors (Rm^2^) was calculated using the *RsqGLM* function in the Bioconductor package *MuMin*.

## Results

### Characteristics of the study participants

Table 1 summarizes the demographic data, lifestyle factors, and health conditions of the study participants in the longitudinal cohort of welders (N=78) and controls (N=96). The median years of welding was ten years at timepoint 1 and 14.5 years at timepoint 2. It should be noted that some controls had done some welding work in the past (N=21 at timepoint 1 and N=22 at timepoint 2). For the welders, their respirable dust concentrations in the working environment decreased with approximately 20% from timepoints 1 to 2 while the exposure to respirable dust (adjusted for respiratory protection equipment) decreased by approximately 30% between timepoints 1 and 2 (table 1). For timepoint 2, the main metals found in the respirable dust were Fe (median=0.41 mg/ m^3^, max=5.37 mg/m^3^) and Mn (median=0.06 mg/m^3^, max=1.54 mg/m^3^) (metal concentrations on the filters were not adjusted for personal breathing protection, N=46). For the controls, the stationary *area-level* of respirable dust concentrations was 0.09 (min–max: 0.02–0.2) mg/m^3^ at timepoint 1 and 0.03 (0.02–0.06) mg/m^3^ at timepoint 2.

**Table 1:** Characteristics of the longitudinal study group of welders and controls measured twice (timepoint 1 and 2). [AI@75=augmentation index, normalized at 75 beats per minutes; CVD=cardiovascular disease; LDL=low-density lipoprotein.]

	Timepoint 1 (2010–2011)				Timepoint 2 (2016–2017)			
	
	Welders (N=78)		Controls (N=96)		Welders (N=78)		Controls (N=96)	
			
	Median (5–95%)	N (%) a,b	Median (5–95%)	N (%) a,b	Median (5–95%)	N (%) a,b	Median (5–95%)	N (%) a,b
Age (years)	43 (23–60)		44 (23–56)		49 (29–66)		50 (29–63)	
Years of welding	10 (1–24.5)		0 (0–11)		14.5 (5–31)		0 (0–11)	
Respirable dust (mg/m^3^) c	1.2 (0.2–4.2)				0.9 (0.1 – 4.2)			
Respirable dust adjusted (mg/m3) d	0.7 (0.2 – 4.2)				0.5 (0.1 – 1.9)			
Cumulative exposure e	5.8 (0.7 – 47.7)				11.0 (1.7 – 36.5)			
Body mass index	27.4 (21.4–34)		27.1 (22–34)		28.8 (22–36)		27.8 (22–35)	
Systolic blood pressure (mmHg)	135 (120–155)		125 (105–145)		129 (112–153)		125 (109–152)	
Diastolic blood pressure (mmHg)	75 (60–91)		70 (55–85)		75 (62–93)		74.5 (62–90)	
Heart rate (beats per minute)	66 (54–81)		65.5 (49 – 81)		64.5 (49–80.5)		66 (52–83)	
Reactive hyperemia index	1.8 (1.3–3.1)		1.8 (1.3–2.8)		1.7 (1.2–2.7)		1.8 (1.3–2.8)	
AI@75	-9.1 (-27.7–12.9)		-7.6 (-28.6-15.4)		-4 (-23.3–24.4)		-5 (-26–19)	
C-reactive protein (mg/L)	1.2 (0.3–4.5)		1.2 (0.3–4.7)		1 (0.3–5.2)		0.98 (0.3–5)	
LDL (mmol/L)	3.1 (1.9–4.7)		3.2 (2–4.7)		3 (1.7–4.2)		3 (2–4.7)	
Homocysteine (µmol/L)	12 (8–16.5)		12 (8–16)		13 (9.8–20)		14 (10–22)	
Country of birth (Sweden)		57 (73)		90 (94)		57 (73)		90 (94)
Education (university or higher)		6 (4)		13 (14)		3 (4)		13 (16)
Residence (large and small cities) f		16 (21)		41 (43)		11 (14)		37 (39)
Hobby exposure to particles g		20 (26)		14 (15)		19 (24)		19 (20)
Smoking history (ever smoked)		32 (41)		34 (35)		32 (41)		37 (39)
Smoking status (currently)								
Non-smoker		76 (97)		93 (97)		75 (96)		91 (95)
Party smoker		2 (3)		3 (0)		1 (1)		2 (2)
Smoker		0 (0)		0 (0)		2 (3)		2 (2)
Current snus use		21 (27)		19 (20)		20 (26)		18 (19)
Alcohol consumption (≥ 3 times/week)		2 (3)		2 (2)		2 (3)		3 (3)
Vegetable intake (≥5 times/week)		47 (60)		62 (65)		42 (54)		69 (72)
Physical activity (moderate/high) h		30 (37)		41 (43)		37 (47)		47 (49)
Personal history of CVD		19 (26)		17 (18)		28 (36)		28 (29)
Family history of CVD		36 (47)		29 (20)		41 (53)		36 (38)

a Categorical variables were categorized as ‘yes’ and ‘no’ unless otherwise stated.

b Percentage calculated relative to the total valid answers.

c Measured or estimated respirable dust (N=78 welders timepoint 1; N=56 welders timepoint 2).

d Adjusted for use of personal respiratory protection equipment.

e Cumulative exposure was calculated from adjusted respirable dust data and reported welding year experience.

f Large and small cities as compared with towns and countryside.

g Exposure to welding fumes, dust, engine exhaust, or engine diesel during leisure activities.

h Physical activity that involves sweating at least once per week and for at least 30 min.

The welders and controls were similar in age, BMI, education, alcohol consumption, snus use, and smoking history. However, the welders were to a larger extent born outside Sweden and lived in small towns when compared with the controls (P<0.005, paired analysis). The median systolic BP decreased from timepoints 1 to 2 in the welders, whereas the diastolic BP increased in the controls from timepoints 1 to 2.

Supplementary table S1 (www.sjweh.fi/show_ abstract.php?abstract_id=3908) shows the self-reported and doctor-diagnosed prevalence of CVD and use of doctors’ prescribed medication, as well as familial history of CVD. Between timepoints 1 and 2, welders reported an increased prevalence of hypertension (welders: 26% at timepoint 1 to 41% at timepoint 2; controls: 15% at timepoint 1 and 26% at timepoint 2) and use of prescribed medication (beta blocker and angiotensin-converting enzyme – ACE – inhibitor) to treat hypertension (welders 12% at timepoint 1 to 21% at timepoint 2; controls: 4% at timepoint 1 and 7% at timepoint 2). There were, however, no significant differences between welders and controls as regards changes in doctor’s diagnosis and medication between timepoints 1 and timepoint 2.

### Cardiovascular disease markers among welders and controls

Table 2 reports the differences between welders and controls for BP, heart rate, endothelial function, and cardiovascular risk biomarkers in plasma (dependent variables) in the study cohort. Over the six-year follow-up period, the systolic BP among welders significantly exceeded that of controls by 5.11 mmHg and the diastolic BP by 3.12 mmHg (model 2). The effect estimates for BP, variance explained by fixed factors, and levels of significance were similar between model 1 only adjusted for age and BMI and the fully adjusted model 2. No significant changes in the markers of endothelial function or risk biomarkers measured in plasma were detected among welders compared with controls over time. In addition, we performed a sensitivity analysis where we excluded the controls who previously welded, and the effect estimates as well as the level of significance were similar to the main analysis.

**Table 2:** Associations between welding and blood pressure, heart rate, endothelial function, and plasma concentrations of biomarkers. [AI@75=augmentation index, normalized at 75 beats per minutes; BP=blood pressure; LDL=low-density lipoprotein; RHI=reactive hyperemia index.]

Outcome	N a	R^2^m (%) b	Beta c (95% CI)	P-value
Model 1^d^				
Systolic BP (mmHg)	348	10	5.25 (2.13–8.38)	0.001
Diastolic BP (mmHg)	348	16	3.02 (0.75–5.29)	0.009
Heart rate	320	4	-0.59 (-3.22–2.03)	0.656
RHI e	327	1	0 (-0.13–0.13)	0.989
AI@75 e	330	29	0.61 (-2.66–3.89)	0.715
omocysteine (µmol/L)	346	2	-0.01 (-1.27–1.26)	0.993
LDL (mmol/L)	348	7	-0.17 (-0.38–0.04)	0.112
C-reactive protein (mg/L)	347	2	-0.14 (-0.95–0.66)	0.730
Model 2 f				
Systolic BP (mmHg)	345	12	5.11 (1.92–8.31)	0.002
Diastolic BP (mmHg)	345	17	3.12 (0.74–5.50)	0.010
Heart rate	317	10	-1.22 (-3.87–1.43)	0.362
RHI e	324	4	-0.01 (-0.15–0.12)	0.844
AI@75 e	327	34	0.09 (-3.23–3.42)	0.958
Homocysteine µmol/L	343	7	-0.24 (-1.53–1.05)	0.718
LDL (mmol/L)	345	9	-0.15 (-0.38–0.07)	0.168
C-reactive protein (mg/L)	344	3	-0.02 (-0.88–0.83)	0.960

a The analysis is based on a maximum of N=348 observations (coming from N=174 individuals; 78 welders and 96 controls, examined six years apart) using linear mixed model analysis. For some outcomes, N is lower than 348 due to missing data, however the number of individuals included in each analysis is 174 since all individuals have complete data for at least one timepoint.

b R^2^m is the variance explained by the fixed factors included in the model.

c Regression coefficient from linear mixed models interpreted as standard deviation difference in outcome between welders and controls.

d Linear mixed-effect model adjusted for age and body-mass index as fixed factors and participant as random factors.

e Measurements of endothelial function were additionally adjusted for drugs effecting EndoPAT as fixed factors.

f Linear mixed-effect model adjusted for age, body-mass index, family history of CVD, physical activity, education status, residence, snus, smoking status, alcohol consumption, dietary intake of vegetables and dietary intake of fish as fixed factors and participant as random factors.

### Exposure–response relationships

The effects of respirable dust, years of welding, and cumulative exposure on cardiovascular outcomes are presented in table 3. The fully adjusted model 2 had in general a higher percentage of variance explained in comparison to models only adjusted for age and BMI. Diastolic BP increased non-significantly by 0.22 mm Hg (P=0.063) with each additional year of welding. Associations between welding years and BP became even stronger when including both welders and controls in the analysis: systolic BP increased 0.25 mmHg with each additional year of welding (95% CI 0.07 – 0.44, P=0.006) and diastolic BP with 0.15 mmHg (95% CI 0.01–0.29, P=0.029). The CRP concentrations increased by 0.86 mg/L with each increment of 1 mg/m^3^ of respirable dust; however, CRP decreased by 0.08 mg/L with each welding year during the follow-up. Homocysteine concentrations in welders decreased by 1.71 μmol/L with every 1 mg/m^3^ increase of respirable dust, whereas cumulative exposure was not associated with any outcome. In order to evaluate non-linear associations, linear mixed models using splines were performed for systolic and diastolic BP (and the same variables as in the main analysis). When comparing the models with and without splines (by ANOVA), there was no significant difference (P=0.169 for systolic BP and P=0.285 for diastolic BP).

**Table 3:** The associations between measurements of exposure to welding (respirable dust, welding years, cumulative exposure) and blood pressure, heart rate, endothelial function as well as plasma concentrations of biomarkers in the longitudinal study of welders. [AI@75=augmentation index, normalized at 75 beats per minutes; CI=confidence interval; LDL=low-density lipoprotein

Exposure /outcome	N a	Model 1 b			Model 2 c		
	
		R^2^m (%) d	Beta ^e^ (95% CI)	P-value	R^2^m (%) d	Beta e (95% CI)	P-value
Respirable dust							
Systolic blood pressure (mmHg)	112	4	0.41 (-1.97–2.79)	0.730	20	-0.34 (-2.78–2.11)	0.782
Diastolic blood pressure (mmHg)	112	7	0.08 (-1.72–1.87)	0.926	12	-0.1 (-2.03–1.82)	0.918
Heart rate	109	5	-0.8 (-2.6–1)	0.381	11	-0.88 (-2.82–1.07)	0.372
Reactive hyperemia index f	110	2	0.03 (-0.1–0.15)	0.695	14	0.05 (-0.08–0.18)	0.464
AI@75 f	110	31	0.2 (-2.69–3.08)	0.892	36	0.46 (-2.6–3.54)	0.765
Homocysteine (µmol/L)	111	4	-0.7 (-2.14–0.73)	0.327	17	-1.71 (-3.2– -0.21)	0.021
LDL (mmol/L)	112	6	-0.03 (-0.17–0.11)	0.660	22	-0.07 (-0.22– 0.08)	0.371
C-reactive protein (mg/L)	112	15	0.67 (0.32–1.01)	<0.001	25	0.86 (0.49–1.22)	<0.001
Welding years							
Systolic blood pressure (mmHg)	156	1	0.07 (-0.25–0.38)	0.642	12	0.07 (-0.23–0.36)	0.623
Diastolic blood pressure (mmHg)	156	8	0.21 (-0.03–0.43)	0.071	11	0.22 (-0.02–0.45)	0.063
Heart rate	153	3	-0.12 (-0.38–0.14)	0.370	8	-0.11 (-0.37–0.15)	0.403
Reactive hyperemia index f	153	3	0 (-0.01–0.02)	0.946	9	0 (-0.01–0.02)	0.750
AI@75 f	153	23	0.23 (-0.14–0.6)	0.212	26	0.24 (-0.12–0.61)	0.192
Homocysteine (µmol/L)	154	4	0.11 (-0.05–0.27)	0.165	12	0.13 (-0.03–0.29)	0.100
LDL (mmol/L)	156	5	0 (-0.02–0.02)	0.782	16	0.01 (-0.02–0.03)	0.609
C-reactive protein (mg/L)	156	11	-0.08 (-0.14– -0.02)	0.006	16	-0.08 (-0.14– -0.02)	0.006
Cumulative exposure							
Systolic blood pressure (mmHg)	112	6	-0.15 (-0.39–0.09)	0.225	21	-0.14 (-0.36–0.08)	0.210
Diastolic blood pressure (mmHg)	112	7	-0.03 (-0.21–0.16)	0.778	12	-0.03 (-0.22–0.16)	0.792
Heart rate	109	5	0.01 (-0.18–0.20)	0.900	10	0.04 (-0.16–0.23)	0.701
Reactive hyperemia index f	110	5	-0.01 (-0.02–0.001)	0.079	14	-0.01 (-0.02–0.004)	0.219
AI@75 f	110	31	-0.1 (-0.37–0.18)	0.488	37	-0.08 (-0.36–0.2)	0.554
Homocysteine (µmol/L)	111	3	-0.02 (-0.16–0.12)	0.811	14	-0.08 (-0.22–0.07)	0.299
LDL (mmol/L)	112	7	-0.01 (-0.02–0.01)	0.423	22	-0.01 (-0.02–0.01)	0.322
C-reactive protein (mg/L)	112	4	0.01 (-0.03–0.04)	0.616	11	0.02 (-0.01–0.06)	0.217

a The analysis is based on welders only using linear mixed model analysis. The analysis is based on a maximum of N=156 observations (coming from N=78 welders) for welding years and a maximum of n=112 observations (coming from N=56 welders) for respirable dust and cumulative exposure. For some outcomes N is lower than maximum due to missing data.

b Linear mixed-effect model adjusted for age and body-mass index as fixed factors and participant as random factors.

c Linear mixed-effect model adjusted for age, body-mass index, family history of CVD, physical activity, education status, residence, snus, smoking status, alcohol consumption, dietary intake of vegetables and dietary intake of fish as fixed factors and participant as random factors.

d R^2^m is the variance explained by the fixed factors included in the model.

e Regression coefficient from linear mixed models interpreted as standard deviation difference in outcome per respirable dust unit increase/numbers of years welding/cumulative exposure unit increase;

f Measurements of endothelial function were additionally adjusted for drugs effecting EndoPAT as fixed factors.

## Discussion

In this study, we found evidence that low-to-moderate exposure to welding fumes increases BP. Although the exposure–response relationship was weak, the diastolic BP seemed to increase with years spent welding. Importantly, this was observed among welders with an average exposure below half the current Swedish occupational exposure limit (OEL) of 2.5 mg/m^3^ for inorganic respirable dust (22). We also uncovered associations between exposure to welding fumes and CRP.

In a previous cross-sectional study of the same welders and controls recruited for cycle 1, we showed that welders have an approximately 5 mm Hg higher systolic and diastolic BP compared with controls (10). In the current study, we validated that putative link between welding and increased BP through a longitudinal experiment design, in which the same welders and controls were revisited six years later. Admittedly, the differences of 5.11 and 3.12 mm Hg respectively for systolic and diastolic BP between the welders and controls in the longitudinal study are rather subtle in relation to known risks of CVD (10, 23). Compared to the previous cross-sectional study on the same cohort (10), in this longitudinal study we observe a similar effect estimate for the BP outcome despite the welders having performed an average four and a half additional years of welding. This could, in part, be related to the BP measurement at timepoint 2 that was more reliable as it included more measurements. In addition, we cannot completely exclude a potential effect of healthy worker selection. Nonetheless, our findings emphasize that the current OEL of 2.5 mg/m^3^ is too high to protect against risk factors for CVD.

For diastolic BP, a weak positive exposure–response relationship with number of years working as a welder (long-term exposure) was revealed, yet no association was found between BP and respirable dust (short-term exposure). It should be noted that respirable dust was evaluated during one day of working and can be considered a measure of acute exposure, which is dynamic but likely a crude proxy for overall level of exposure. This could potentially result in a misclassification of exposure to respirable dust. Instead, respirable dust was associated with CVD-related serum markers CRP and homocysteine in our study, for which higher levels in all have been linked to greater CVD risk (24–26), though Mendelian randomization studies have questioned the role of CRP (27). Increased CRP was positively associated with respirable dust in this longitudinal study suggesting an increased risk of CVD, whereas homocysteine was negatively associated with respirable dust. CRP is an acute phase protein involved in systemic inflammation, and its range of values that define different risk categories are quite narrow (low risk ≤1.0 mg/L, intermediate risk=1.0–3.0 mg/L, and high risk ≥3.0 mg/L (28);). Moreover, CRP has also been associated with PM2.5 exposure in several recent studies (29–31), pointing to systemic inflammation as the underlying mechanism between PM exposure and CVD. Yet, since respirable dust was not measured on the same day as blood sampling, its presented associations with plasma markers should be interpreted cautiously as the levels of exposure can vary highly between working days.

In both timepoints 1 and 2 of this study, the median respirable dust levels (both in the working environment and after correction for personal respiratory protective equipment) fell below the Swedish OEL of 2.5 mg/ m3 established in 2018 (22). Still, we found relationships between welding and BP and between respirable dust and CVD risk markers in plasma. Together, these results indicate that Sweden’s OEL remains too high to protect welders from CVD-related health effects from occupational exposure, particularly for those individuals working long-term in welding. It should be noted that the levels of respirable dust were lower in timepoint 2 compared with timepoint 1. We speculate that this may be due to the recommendations for improvement of the work environment that we gave along with the results for respirable dust to each company after timepoint 1. Each company participating in the study received a report about their respirable dust level (welding companies) or stationary particle measurements (control companies) where the levels were related to the Swedish OEL and suggestions of actions were given to reduce the exposure levels (if above the OEL). Apart from that, we also sent out a short report to each worker that participated at the first timepoint about the results of that study (cross-sectional analyses of the cohort).

Although the median respirable dust in the air was below the OEL, the median levels of respirable Mn were above the OEL for Mn [0.05 mg/m^3^ (22)] at timepoint 2, and concentrations nearly 30 times the OEL were detected (the Mn concentrations on the filters were not adjusted for personal breathing protection). The mild steel used by the welders in this study was the most commonly used type in terms of its Fe and Mn contents, and our results suggest that many welders in Sweden are exposed to excess levels of respirable Mn. However, since Mn exposure from welding fumes is known to cause neurological effects (32, 33), but is not suspected to be a risk factor for CVD, Mn exposure was not evaluated in relation to CVD-related outcomes in this study.

We must note that median systolic BP among welders in the longitudinal study group was lower at timepoint 2 than timepoint 1, which is an unexpected result given the higher age – and longer working history in welding – of participants at timepoint 2. However, the reported hypertension increased among welders increasing from timepoints 1 to 2 and some of the reduction in systolic BP may be due to increased use of medicine against hypertension. Another explanation for the lower median BP at timepoint 2 is that measurements were taken differently between cycles; at timepoint 1 one measurement was used whereas at timepoint 2 an average of two measurements were used. Thus, the latter data should more accurately convey resting BP. Nevertheless, since these measurements were identical between welders and controls within each cycle, they should be valid for comparison between groups over time.

There are both strengths and limitations to this study. Its strengths include the longitudinal study design wherein the same repeated measurements were performed on the same individuals six years apart. Moreover, many influential factors were considered in this study, and we have excluded two important confounders of CVD by recruiting only non-smoking welders and controls at baseline and assessing workloads of welders and controls (found to be similar). However, we cannot completely exclude unmeasured differences between the welders and controls, which may explain discrepancies between results obtained in welders and controls and the exposure–response associations in welders only. Furthermore, the exposure to welding fumes was robustly characterized through individual respirable dust measurements performed for many welders. However, it should be noted that respirable dust is performed during one day of working and can be considered a measure of acute exposure and a crude measure of level of exposure. This could potentially result in a misclassification of exposure to respirable dust. Additional limitations include that recommended conditions for EndoPAT measurements [ie, fasting (a minimum of 4–8 hours), avoiding smoking, snus, or consuming caffeine for ≥8 hours before measurements, as well as not taking CVD-related medication on the testing day] were not feasible at workplaces during the daytime. Moreover, follow-up measurements of participants at the same time of the day could not be ensured, and, as mentioned already, the conditions for BP measurements were not identical between timepoints.

In conclusion, this study has supported previously documented associations between welding and increased BP and thereby strengthens the link between welding and CVD. Furthermore, the results suggest the occupational exposure of Swedish welders persists at levels that can cause biological effects and the current OEL for respirable dust of 2.5 mg/m^3^ is too high to adequately protect welders from work-related CVD.

## Funding

The Swedish Council for Working Life and Social Research (Forte), the Karolinska Institute, and the Division of Occupational and Environmental Medicine, Lund University Hospital supported this study. This project is a part of the EU-Cost Action, CA 15129 (DiMoPEx), which is supported by the EU Framework Program Horizon 2020.

## Supplementary material

Supplementary material
