# Supplementary material for: Effect of welding fumes on the cardiovascular system: a six-year longitudinal study
Source: Scand J Work Environ Health. 2020 Dec 16;47(1):52–61. doi: 10.5271/sjweh.3908 (PMC7801138; doi:10.5271/sjweh.3908)
Supplement: Supplementary material [file SJWEH-47-52-S001.pdf]

# Effect of welding fumes on the cardiovascular system: a six-year longitudinal study[1]

by Tahir Taj, PhD, Anda R Gliga, PhD, Maria Hedmer, PhD, Karin Wahlberg, PhD, Eva Assarsson, RN, Thomas Lundh, PhD, Håkan Tinnerberg, PhD, Maria Albin, PhD, Karin Broberg, PhD[2]

1. *Supplementary material*
2. *Correspondence to: Karin Broberg, Institute of Environmental Medicine, Karolinska Institutet, Stockholm, Sweden. [E-mail: karin.broberg@ki.se]*

Supplementary Table S1. Self-reported cardiovascular disease (CVD), medication for CVD, and family history of CVD in welders (n=78) and controls (n=96) measured 6 years apart.

| | Timepoint 1 2010-2011 | | Timepoint 2 2016-2017 | |
|---|---|---|---|---|
| | Welders | Controls | Welders | Controls |
| *Self-Reported CVD n (%)* | | | | |
| Myocardial infarction | 0 (0) | 0 (0) | 0 (0) | 0 (0) |
| Angina pectoris | 7 (9) | 7 (7) | 10 (13) | 5 (5) |
| Hypertension | 20 (26) | 14 (15) | 32 (41) | 25 (26) |
| Stroke | 0 (0) | 0 (0) | 2 (3) | 0 (0) |
| Blood clot | 0 (0) | 2 (2) | 2 (3) | 4 (4) |
| Other heart diseases | 0 (0) | 2 (2) | 2 (3) | 6 (6) |
| *Medication for CVD n (%)* | | | | |
| Beta blocker | 2 (3) | 2 (2) | 7 (9) | 1 (1) |
| ACE inhibitor | 7 (9) | 2 (2) | 9 (12) | 6 (6) |
| Calcium channel blocker | 0 (0) | 0 (0) | 1 (1) | 1 (1) |
| Statin | 2 (3) | 0 (0) | 5 (6) | 8 (8) |
| Serotonin receptor inhibitor | 1(1) | 0 (0) | 0 (0) | 0 (0) |
| *Family history of CVD n (%)* | | | | |
| Myocardial infarction | 17 (22) | 19 (20) | 23 (29) | 23 (24) |
| Stroke | 13 (17) | 12 (13) | 14 (18) | 13 (14) |
| Hypertension | 51 (65) | 59 (61) | 56 (72) | 72 (75) |
